# Early predictors of unfavorable outcomes in pediatric acute respiratory failure

**DOI:** 10.1186/s40560-024-00763-x

**Published:** 2024-12-02

**Authors:** Shinya Miura, Nobuaki Michihata, Toshiaki Isogai, Hiroki Matsui, Kiyohide Fushimi, Hideo Yasunaga

**Affiliations:** 1 Department of Pediatrics, St. Marianna University School of Medicine, 2 Chome-16-1 Sugao, Miyamae Ward, Kawasaki, Kanagawa Japan; 2 Cancer Prevention Center, Chiba Cancer Center Research Institute, Chiba, Japan; 3 Department of Health Services Research, Graduate School of Medicine, The University of Tokyo, Tokyo, Japan; 4 Department of Cardiology, Tokyo Metropolitan Tama Medical Center, Tokyo, Japan; 5 Department of Clinical Epidemiology and Health Economics, School of Public Health, The University of Tokyo, Tokyo, Japan; 6 Department of Health Policy and Informatics, Tokyo Medical and Dental University Graduate School, Tokyo, Japan

**Keywords:** Respiratory failure, Epidemiology, Prognosis, Comorbidity, Children, Pediatric

## Abstract

**Objective:**

Acute respiratory failure is a leading cause of critical illness in children. However, patient outcomes and early predictors of unfavorable outcomes are not well understood. This study aimed to describe composite unfavorable outcomes, defined as in-hospital death or discharge with new comorbidities, and to identify early predictors in children with acute respiratory failure in acute care hospitals.

**Design:**

Retrospective cohort study using a national inpatient database in Japan.

**Setting:**

All acute care hospitals registered in the database.

**Patients:**

This study included children under 20 years of age who were admitted with acute respiratory diseases between July 2010 and March 2022 and received ventilatory support within the first three days of hospitalization.

**Intervention:**

None.

**Measurements and main results:**

Among 29,362 eligible children, the median age was 1.2 (interquartile range, 0.3–3.7) years and 28.8% had underlying conditions. The highest level of ventilatory support within the first three days was invasive ventilation (69.4%), noninvasive ventilation (1.0%), and high-flow nasal cannula (29.7%). Respiratory diagnoses included pneumonia (58.6%), bronchiolitis (29.0%), and asthma (11.1%). Among these children, 669 (2.3%) died and 1994 (6.8%) were discharged with new comorbidities, resulting in 2663 (9.1%) children experiencing unfavorable outcomes. In the logistic regression model, older age, underlying conditions, pneumonia, and low hospital volume were associated with unfavorable outcomes after adjusting for covariates.

**Conclusions:**

A significant proportion of pediatric patients with acute respiratory failure experienced unfavorable outcomes, warranting future efforts to improve acute care services for at-risk children. Early predictors identified from national database analyses could inform risk stratification and optimize the provision of acute care services for vulnerable pediatric patients.

**Supplementary Information:**

The online version contains supplementary material available at 10.1186/s40560-024-00763-x.

## Introduction

Acute respiratory failure is a leading cause of critical illnesses in children worldwide [1, 2]. Despite advances in medical care that have reduced mortality, it remains a significant public health issue, with a substantial proportion (10–25%) of hospital survivors developing new comorbidities [3–9].

Understanding epidemiological information, including the prognosis of children with acute respiratory failure and early predictors of unfavorable outcomes, is essential for coordinating acute care services. However, existing studies often focused on specific respiratory diagnoses or were limited to certain types of healthcare facilities, resulting in a lack of comprehensive understanding of the broader pediatric population affected by acute respiratory failure [3–8]. In addition, there is limited knowledge about the early predictors of unfavorable outcomes, such as in-hospital death and discharge with new comorbidities, in the general cohort of pediatric respiratory failure [8]. Therefore, we aimed to describe the prognosis of children with acute respiratory failure admitted to acute care hospitals in Japan and to identify early predictors of unfavorable outcomes.

## Methods

### Study design and participants

We performed a retrospective cohort study using data from the Diagnosis Procedure Combination database in Japan. This database, based on the medical fee reimbursement system, contains discharge abstracts and claims data from over 1000 acute care hospitals [10]. This dataset represents more than half of Japan’s annual inpatient admissions, covering approximately seven million cases per year, including 24 of the 27 hospitals with PICUs. It includes patient characteristics, Japan Coma Scale scores [11], diagnoses, comorbidities, treatments, administrative information, and discharge outcomes. Diagnoses were recorded using International Classification of Diseases 10th Revision (ICD-10) codes. Studies validating the Diagnosis Procedure Combination data against medical chart reviews have shown a specificity of 93.2% and a sensitivity of 78.9% for primary diagnoses, with both specificity and sensitivity for recorded procedures exceeding 90% [10, 12]. The Institutional Review Board of the University of Tokyo approved this study (approval number: 3501-(5); May 19, 2021), and the requirement for informed consent was waived because of the use of anonymized data.

We included children aged < 20 years who were admitted to acute care hospitals with acute respiratory diseases under primary or admission diagnoses between July 2010 and March 2022, and who received ventilatory support, including invasive ventilation, non-invasive ventilation, and high-flow nasal cannula, within the first three days of hospitalization. The study included a range of respiratory diseases categorized as pneumonia (ICD-10, J09-J18, J69.0, J80, J96.0, J96.9, U04.9, U07.1), bronchiolitis (J20-J22, J40), asthma (J44-J46), and other diseases such as croup (J05), tracheitis (J04.1, J04.2), pertussis (A37), and lung abscess (J85, J86).

The following exclusion criteria were applied: (i) infants hospitalized continuously from birth, (ii) patients transferred to other hospitals on invasive ventilation within 5 days of admission, and (iii) previous enrolment during the study period.

### Outcomes and covariates

The primary outcome was a composite of unfavorable outcomes, defined as in-hospital death or discharge with new comorbidities, specifically tracheostomy, home ventilation or oxygen therapy, tube feeding at discharge or the day before, gastrostomy during hospitalization, worsened neurological status based on the Japan Coma Scale score at discharge, and renal failure, defined as the need for renal replacement therapy within three days of discharge.

Other variables included patient characteristics and clinical data, such as age, sex, diagnoses, underlying conditions, clinical interventions, and administrative information. Microbiological diagnoses were categorized as viral, bacterial, aspiration, COVID-19, or no detection. We classified the underlying conditions according to complex chronic conditions using ICD-10 codes, and the presence of underlying conditions was defined based on the presence of any of the following conditions: neurological/neuromuscular, cardiovascular, respiratory, congenital/genetic, premature/neonatal, hematological/immunological, malignant, or transplant conditions [13]. Impaired consciousness was assessed using the Japan Coma Scale and categorized into three groups: severe, moderate, and mild, corresponding to scores of approximately 3–8, 9–13, and 14–15 on the Glasgow Coma Scale, respectively [11, 14, 15]. Patients were considered to have acute liver failure if their admission diagnosis included ICD-10 codes K72.0, K72.9, or K76.3, and thrombocytopenia/coagulopathy if they included ICD-10 codes D65, D68.9, D69.5, or D69.6 [16]. Hospital volume was defined as the number of eligible children in each hospital during the study period, and was categorized into tertiles (low, medium, and high). According to the Japanese administrative claims system, accredited intensive care units (ICUs) include PICUs, neonatal ICUs, general ICUs, emergency ICUs, and intermediate care units.

### Statistical analyses

We used multivariable logistic regression models to identify early predictors of composite unfavorable outcomes and in-hospital mortality, using generalized estimating equations to adjust for clustering within hospitals. As studies reporting predictive factors for unfavorable outcomes in pediatric respiratory failure are scarce, studies including critically ill children were also referred to select study covariates. The study covariates included patient characteristics (age and underlying conditions), respiratory diagnostic category [8], therapies provided within three days of hospitalization (invasive ventilation, vasoactive drugs, corticosteroids, extracorporeal membrane oxygenation, renal replacement therapy, cardiopulmonary resuscitation, and surgery), and organ dysfunction on admission (level of consciousness, acute liver failure, thrombocytopenia/coagulopathy) [17–19]. We also included microbiological diagnoses (viral, bacterial, aspiration, COVID-19, no detection), transport from other hospitals, admission to accredited ICUs within the first 3 days of hospitalization, and hospital volume to adjust for pathogenic and administrative variables. The variance inflation factor was calculated for each covariate to evaluate the multicollinearity of the study covariates. To ensure the robustness of the primary analysis, we performed sensitivity analyses by (i) including only patients requiring invasive ventilation, (ii) excluding patients with bronchiolitis, and (iii) excluding patients with underlying neurological/neuromuscular conditions. All statistical analyses were performed using STATA 17 (StataCorp LLC, College Station, TX, USA).

## Results

### Patient characteristics and therapies

**Fig. 1:**
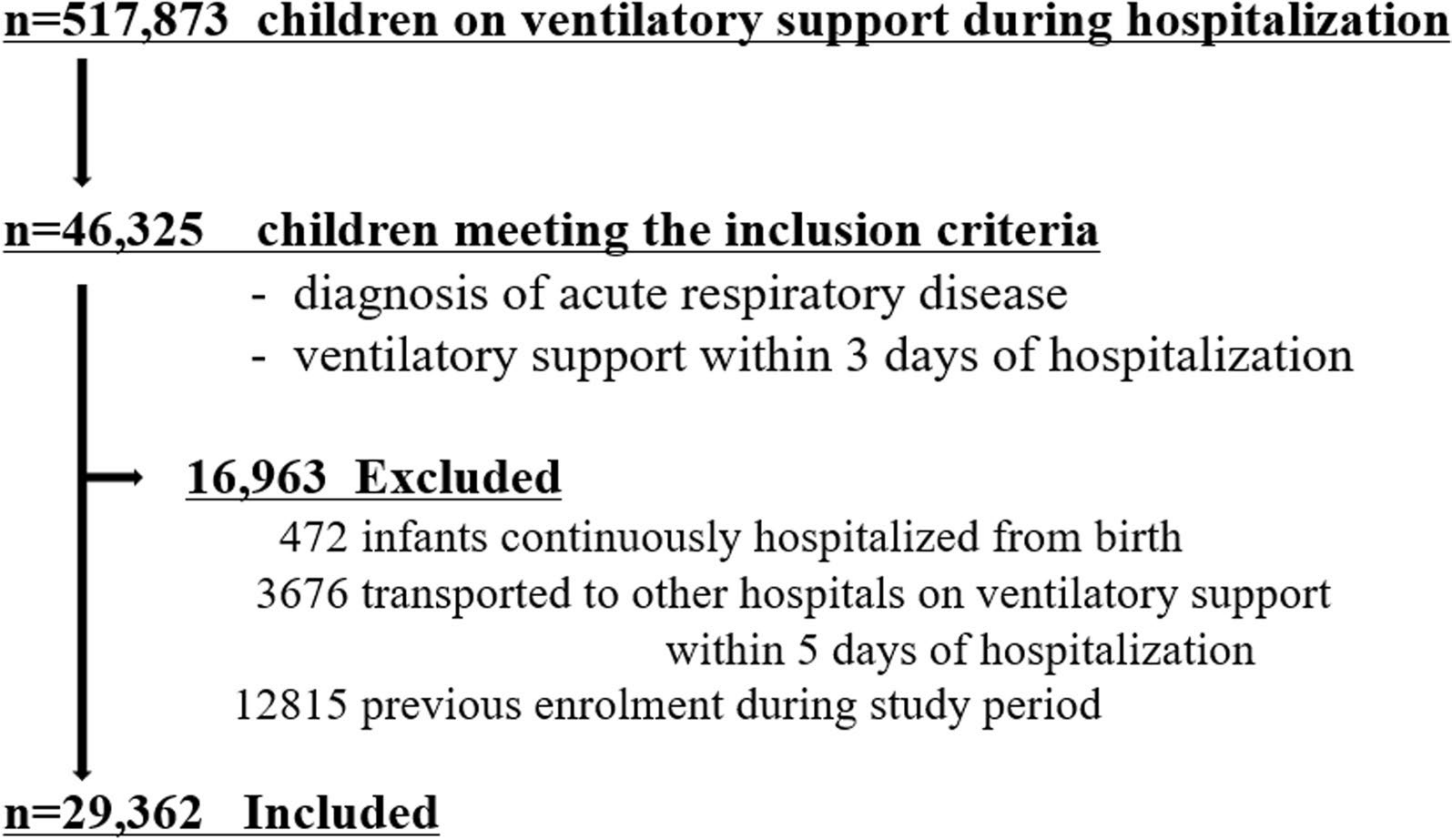
Patient flow

**Fig. 2:**
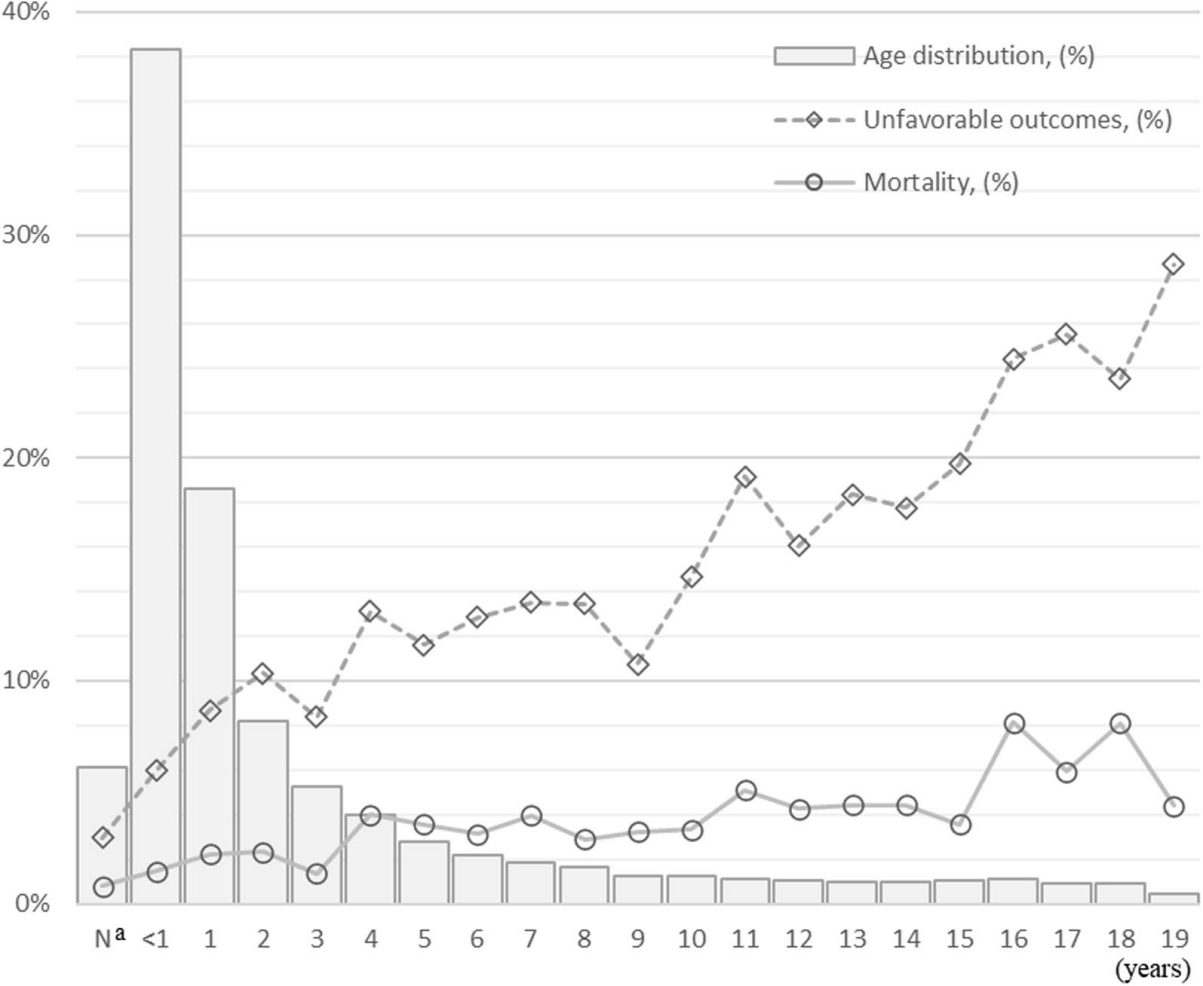
Patient distribution and outcomes by age (n = 29,362). N^a^ indicates neonates of ≤ 28 days old. Unfavorable outcomes included in-hospital death, discharge with new comorbidities—specifically, tracheostomy, home ventilation or oxygen therapy, tube feeding at discharge or the day before, gastrostomy during hospitalization, worsened neurological status at discharge, and renal failure

We identified 29,362 children with acute respiratory failure (Fig. 1). The median age was 1.2 (interquartile range (IQR), 0.3–3.7) years. Infants (29 days to < 1 year) accounted for 38.3% of the patients (n = 11,258), followed by 1-year-olds (18.6%; n = 5470), and 2-year-olds (8.2%; n = 2,398) (Fig. 2). One-third of the children (28.8%; n = 8446) had ≥ 1 underlying condition, with neurological/neuromuscular (13.4%; n = 3,926) as the most common, followed by cardiovascular (7.5%; n = 2194), congenital/genetic (6.4%; n = 1880), respiratory (6.0%; n = 1,753), hematological/immunological/malignancy/transplantation (2.4%; n = 715), and premature/neonatal (1.3%; n = 395) conditions. The main respiratory diagnoses were pneumonia (58.6%, n = 17,201), bronchiolitis (29.0%, n = 8506), and asthma (11.1%, n = 3260). Among all children, 18.7% (n = 5496) were transported from other hospitals and 29.0% (n = 8521) were admitted to accredited ICUs within the first three days of hospitalization (Table 1). The 29,362 children included in this study were treated at 666 hospitals, with a median hospital volume of 105 (IQR, 51–319).

**Table 1:** Characteristics, therapies and outcomes of children with acute respiratory failure (n = 29,362)

Variables	n	(%)
Age, years, median (IQR)	1.2	(0.3–3.7)
< 1 year	13,057	(44.5)
1–5 years	11,399	(38.8)
6–19 years	4906	(16.7)
Male	16,563	(56.4)
Underlying conditions, any	8446	(28.8)
Neurological/neuromuscular	3926	(13.4)
Cardiovascular	2194	(7.5)
Respiratory	1753	(6.0)
Congenital/genetic	1880	(6.4)
Premature/neonatal	395	(1.3)
Hematological/immunological/malignancy/transplantation	715	(2.4)
Diagnostic category		
Pneumonia	17,201	(58.6)
Bronchiolitis	8506	(29.0)
Asthma	3260	(11.1)
Others	395	(1.3)
Pathogen		
Viral	12,135	(41.3)
Bacterial	3826	(13.0)
Aspiration	785	(2.7)
COVID-19	48	(0.2)
No detection	12,568	(42.8)
Organ dysfunction on admission		
Impaired consciousness		
No-mild	26,409	(89.9)
Moderate	859	(2.9)
Severe	2094	(7.1)
Acute liver failure	12	(0.0)
Thrombocytopenia/coagulopathy	276	(0.9)
Transported from other hospitals	5496	(18.7)
Admitted to accredited ICUs	8521	(29.0)
Hospital volume, cases, median (IQR)	105	(51–319)
Length of ventilatory support, days, median (IQR)	5	(3–8)
Length of invasive ventilation, days, median (IQR) ^a^	6	(3–9)
Length of hospital stay, days, median (IQR)	9	(7–14)
In-hospital death	669	(2.3)
Discharged with comorbidities	1994	(6.8)

Data are presented as n (%) unless otherwise indicated

*COVID-19*, coronavirus disease. 2019; *IQR*, interquartile range; *ICU*, intensive care unit

^a^Length of invasive ventilation in 20,365 children mechanically ventilated within the first three days of hospitalization

Regarding the highest level of ventilatory support, 69.4% (n = 20,365) received invasive ventilation, 1.0% (n = 289) received noninvasive ventilation, and 29.7% (n = 8708) received high-flow nasal cannula within the first three days of hospitalization. Renal replacement therapy, extracorporeal membrane oxygenation, surgery, and cardiopulmonary resuscitation were provided to 0.3% (n = 95), 0.3% (n = 87), 0.3% (n = 91), and 1.5% (n = 447) of the children, respectively. Vasoactive drugs and corticosteroids were administered to 6.9% (n = 2023) and 41.3% (n = 12,136) of patients, respectively (Table 2).

**Table 2:** Therapies within three days of hospitalization (n = 29,362)

Therapies	n	(%)
Ventilatory support^a^		
Invasive ventilation	20,365	(69.4)
Non-invasive ventilation	289	(1.0)
HFNC	8708	(29.7)
Other intervention		
Renal replacement therapy	95	(0.3)
ECMO	87	(0.3)
Surgery	91	(0.3)
Cardiopulmonary resuscitation	447	(1.5)
Medication		
Vasoactive drug	2023	(6.9)
Corticosteroid	12,136	(41.3)
Immunoglobulin	1054	(3.6)
Surfactant	67	(0.2)
Inhaled nitric oxide	42	(0.1)

HFNC, high-flow nasal cannula; ECMO, extracorporeal membrane oxygenation

^a^ categorized by the highest level of ventilatory support within the first three days of hospitalization

### Mortality and unfavorable outcomes

Among all children, 2.3% (n = 669) died, and 6.8% (n = 1994) were discharged with new comorbidities, resulting in 9.1% (n = 2663) of children experiencing unfavorable outcomes. A detailed analysis of these children with unfavorable outcomes showed that 3.5% (n = 1039) were discharged with respiratory impairment, including tracheostomy (2.1%; n = 603), home ventilation (1.2%; n = 348), and home oxygen therapy (0.9%; n = 250). Other comorbidities included tube feeding/gastrostomy (2.9%, n = 852), worsened neurological status (1.9%, n = 563), and renal failure at discharge (0.003%, n = 1).

Regarding respiratory diagnoses, almost all comorbidities were most common in patients with pneumonia, with tracheostomy accounting for 3.2% of this diagnostic cohort (Table 3). Based on the highest level of ventilatory support within the first three days of hospitalization, unfavorable outcomes occurred in 11.8% (n = 2393) of children on invasive ventilation, 6.9% (n = 20) of children on noninvasive ventilation, and 2.9% (n = 250) of children on high-flow nasal cannula. The in-hospital mortality rate was 3.2% (n = 652) in children on invasive ventilation, 0% (n = 0) in children on non-invasive ventilation, and 0.2% (n = 17) in children with high-flow nasal cannulas (Table 4).

**Table 3:** Details of unfavorable outcomes by diagnostic category

	All	Pneumonia	Bronchiolitis/Asthma	Others
	n = 29,362	n = 17,201	n = 11,766	n = 395
Any unfavorable outcome, n, (%)	2,663 (9.1)	2,234 (13.0)	408 (3.5)	21 (5.3)
In-hospital death, n, (%)	669 (2.3)	602 (3.5)	62 (0.5)	5 (1.3)
Respiratory impairment, n, (%)	1039 (3.5)	929 (5.4)	99 (0.8)	11 (2.8)
Tracheostomy, n, (%)	603 (2.1)	559 (3.2)	34 (0.3)	10 (2.5)
Home ventilation, n, (%)	348 (1.2)	310 (1.8)	35 (0.3)	3 (0.8)
Home oxygen therapy, n, (%)	250 (0.9)	212 (1.2)	37 (0.3)	1 (0.3)
Tube feeding/gastrostomy, n, (%)	852 (2.9)	732 (4.3)	113 (1.0)	7 (1.8)
Worsened neurological status, n, (%)	563 (1.9)	389 (2.3)	171 (1.5)	3 (0.8)
Renal failure, n, (%)	1 (0.0)	1 (0.0)	0	0

Unfavorable outcomes included in-hospital death, discharge with new comorbidities—specifically, tracheostomy, home ventilation or oxygen therapy, tube feeding at discharge or the day before, gastrostomy during hospitalization, worsened neurological status at discharge, and renal failure

**Table 4:** Details of unfavorable outcomes by the highest level of ventilatory support within 3 days of hospitalization

	All		Invasive ventilation		Non-invasive ventilation		High-flow nasal cannula	
	n = 29,362		n = 20,365		n = 289		n = 8,708	
Any unfavorable outcome, n, (%)	2,663	(9.1)	2,393	(11.8)	20	(6.9)	250	(2.9)
In-hospital death, n, (%)	669	(2.3)	652	(3.2)	0	0.0	17	(0.2)
Respiratory impairment, n, (%)	1,039	(4.2)	975	(6.2)	8	(2.8)	56	(0.6)
Tracheostomy, n, (%)	603	(2.5)	581	(3.7)	1	(0.3)	21	(0.2)
Home ventilation, n, (%)	348	(1.4)	322	(2.1)	4	(1.4)	22	(0.3)
Home oxygen therapy, n, (%)	250	(1.0)	230	(1.5)	3	(1.0)	17	(0.2)
Tube feeding, n, (%)	852	(3.5)	762	(4.9)	10	(3.5)	80	(0.9)
Worsened neurological status, n, (%)	563	(2.3)	433	(2.8)	7	(2.4)	123	(1.4)
Renal failure, n, (%)	1	(0.0)	1	(0.0)	0		0	

Unfavorable outcomes included in-hospital death, discharge with new comorbidities—specifically, tracheostomy, home ventilation or oxygen therapy, tube feeding at discharge or the day before, gastrostomy during hospitalization, worsened neurological status at discharge, and renal failure

### Early predictors for unfavorable outcomes

In the logistic regression model, unfavorable outcomes were associated with older age, underlying conditions, pneumonia diagnosis, no pathogenic diagnoses, thrombocytopenia/coagulopathy, and low hospital volume after adjusting for therapies and care provided within three days of hospitalization (Table 5). Impaired consciousness on admission was associated with in-hospital death, but did not significantly affect the proportion of unfavorable outcomes. Admission to accredited ICUs within the first three days of hospitalization was associated with a reduced proportion of in-hospital deaths but an increased proportion of unfavorable outcomes. All variance inflation factors for each covariate were less than 2, indicating no significant multicollinearity (Suppl. Table 1).

**Table 5:** Multivariable analyses for factors associated with composite unfavorable outcomes and death (n = 29,362)

Variables	Unfavorable outcomes			In-hospital death		
	Odds ratio (95% CI)		*p*	Odds ratio (95% CI)		*p*
Age						
< 1 year	Reference			Reference		
1–5 years	1.33	(1.13–1.56)	< 0.01	1.28	(0.98–1.67)	0.07
6–19 years	1.50	(1.28–1.75)	< 0.01	1.10	(0.81–1.49)	0.55
Underlying conditions	2.69	(2.41–2.99)	< 0.01	2.02	(1.61–2.53)	< 0.01
Diagnostic category						
Bronchiolitis	Reference			Reference		
Pneumonia	1.97	(1.72–2.25)	< 0.01	2.41	(1.70–3.41)	< 0.01
Asthma	0.89	(0.69–1.14)	0.35	0.96	(0.54–1.69)	0.88
Others	0.68	(0.43–1.08)	0.11	0.65	(0.23–1.78)	0.40
Pathogen						
Viral	Reference			Reference		
Bacterial	1.15	(0.94–1.41)	0.17	1.12	(0.78–1.61)	0.55
Aspiration	0.86	(0.60–1.23)	0.41	0.71	(0.29–1.75)	0.46
COVID-19	1.00	(0.39–2.51)	0.99	1.67	(0.31–9.07)	0.55
No detection	1.68	(1.46–1.93)	< 0.01	1.70	(1.28–2.28)	< 0.01
Organ dysfunction on admission						
Impaired consciousness						
No-mild	Reference			Reference		
Moderate	1.18	(0.96–1.44)	0.11	2.02	(1.36–2.99)	< 0.01
Severe	1.02	(0.89–1.17)	0.76	2.33	(1.80–3.00)	< 0.01
Acute liver failure	2.85	(0.60–13.44)	0.19	1.45	(0.32–6.51)	0.63
Thrombocytopenia/coagulopathy	1.97	(1.78–3.31)	< 0.01	2.50	(1.48–4.25)	< 0.01
Therapies within 3 days of admission						
Invasive ventilation	1.43	(1.21–1.69)	< 0.01	3.45	(1.97–6.06)	< 0.01
Renal replacement therapy	2.20	(1.28–3.78)	< 0.01	3.40	(1.70–6.48)	< 0.01
ECMO	1.06	(0.55–2.03)	0.87	0.60	(0.25–1.42)	0.25
Surgery	3.83	(2.27–6.44)	< 0.01	0.83	(0.23–2.98)	0.77
Cardiopulmonary resuscitation	12.87	(9.64–17.18)	< 0.01	23.14	(16.93–31.62)	< 0.01
Vasoactive drug	2.61	(2.23–3.07)	< 0.01	9.25	(7.17–11.93)	< 0.01
Corticosteroid	1.01	(0.91–1.13)	0.81	0.82	(0.65–1.05)	0.12
Transported from other hospitals	1.15	(1.00–1.32)	0.05	1.05	(0.82–1.36)	0.69
Admitted to accredited ICUs	1.59	(1.39–1.81)	< 0.01	0.63	(0.48–0.82)	< 0.01
Hospital volume						
1–58	1.47	(1.14–1.91)	< 0.01	2.63	(1.97–3.51)	< 0.01
59–144	1.21	(0.93–1.81)	0.16	1.90	(1.40–2.58)	< 0.01
145–752	Reference			Reference		

*CI*, confidence interval; *COVID-19*, coronavirus disease. 2019; *ECMO*, extracorporeal membrane oxygenation; *ICU*, intensive care unit

Unfavorable outcomes included in-hospital death, discharge with new comorbidities—specifically, tracheostomy, home ventilation or oxygen therapy, tube feeding at discharge or the day before, gastrostomy during hospitalization, worsened neurological status at discharge, and renal failure

### Sensitivity and subgroup analyses

In the sensitivity analyses (i) including only children requiring invasive ventilation, (ii) excluding children with bronchiolitis, and (iii) excluding children with underlying neurological/neuromuscular conditions, we confirmed similar results with regard to early predictors and outcomes by age (Suppl Table 2–4 and Suppl Fig. 1).

## Discussion

This study found an unfavorable outcome prevalence of 9.1%, including an in-hospital mortality rate of 2.3%, and identified early predictors in children with acute respiratory failure in acute-care hospitals. To our knowledge, this is the first large-scale study to describe both mortality and new comorbidities, along with their predictive factors, among children with a wide spectrum of respiratory diseases in a broad range of hospitals. Therefore, we believe that our findings can be generalized to a general cohort of pediatric respiratory failure patients admitted to acute care hospitals in high-income countries.

Epidemiological data on the outcomes of patients with acute respiratory failure are limited. In a study of invasively ventilated children with acute respiratory failure, the in-hospital mortality rate was 4.5% [20]. In the follow-up of the same cohort, a decreased functional status was observed in 9.4% of the hospital survivors [3]. In another study of children mechanically ventilated for bronchiolitis, functional decline was observed in 12% of patients at 6 months after PICU discharge [21]. The lower mortality rate in this study may be due to the inclusion of patients with a wider range of severity, including those on a high-flow nasal cannula or non-invasive ventilation, and milder cases treated in acute care hospitals outside PICUs. Conversely, a significant proportion of patients experienced unfavorable outcomes, consistent with previous reports, highlighting the significant social impact on quality of life given the large number of pediatric patients presenting with acute respiratory failure.

This study identified older age, underlying conditions, pneumonia diagnosis, and low hospital volume as early predictors of unfavorable outcomes. In an analysis of invasively ventilated children with acute respiratory failure, health-related quality of life was predominantly impaired in older children (age ≥ 5 years) [8]. Furthermore, in a general cohort of critically ill children, recovery after critical illness was limited in older children [22]. Similarly, our analysis showed an increased risk of unfavorable outcomes in older children, suggesting a need for improved acute care services for older children and adolescents, a group typically underrepresented in both PICUs and adult ICUs.

With regard to underlying conditions, previous studies in a general cohort of critically ill children have reported conflicting results, with some suggesting that underlying conditions place patients at a greater risk of critical illness and further deterioration of their functional status [23, 24], whereas others report greater functional decline in children with a normal baseline status because they have more skills and abilities to lose [9, 25]. This discrepancy may be due to the definition of the underlying conditions and outcome measurement. Previous studies have examined the association between baseline functional scores and changes at discharge. However, these findings may be limited by statistical bias: children with impaired baseline function have less potential for further decline at discharge, which may introduce a “protective” bias, making impaired baseline function appear less associated with unfavorable outcomes [9, 24]. Furthermore, in some studies, children were classified as impaired if they had a decline of one or more points in the Pediatric Overall Performance Category or Pediatric Cerebral Performance Category at discharge compared with baseline. This approach raises concerns about the inclusion of children with milder comorbidities, which may affect the clinical significance of the results [24, 25]. In contrast, our study defined unfavorable outcomes based on a composite of factors reflecting a significantly reduced quality of life, and the covariate for the underlying condition was based on an established methodology [13] and was not highly collinear with the outcome, ensuring a more distinct and clinically meaningful approach. In our study, children with underlying conditions had more than twice the odds of unfavorable outcomes and mortality, suggesting that this vulnerable cohort may benefit from receiving acute care in high-volume hospitals that are generally better staffed and equipped to provide both specialized acute care and comprehensive follow-up.

We also found that 13% of the children diagnosed with pneumonia had unfavorable outcomes, with a diagnosis of pneumonia being an independent risk factor after adjustment. Although we cannot conclude causality, in the present dataset, a diagnosis of pneumonia may reflect the progression of respiratory diseases with evolving opacities on chest radiography. Therefore, the prognostic risk in children with other respiratory categories, such as bronchiolitis, should not be underestimated in light of a study that reported a decreased functional status in 12% of children invasively ventilated for bronchiolitis [21]. In addition, due to the nature of this database, which is based on the Japanese medical fee reimbursement system, pneumonia diagnoses in this dataset may not necessarily correspond to clinical diagnoses. Therefore, comparisons of outcomes between respiratory diagnosis categories should be interpreted with caution.

In line with previous research, low hospital volume was associated with an increased risk of unfavorable outcomes [26]. This association may be due not only to better staffing and equipment in high-volume hospitals but also to factors such as cumulative experience, structured training programs, standardized protocols, multidisciplinary management, and greater access to advanced technology [27]. The variation in outcomes by hospital volume has highlighted the need for strategies to reduce unfavorable outcomes in children with acute respiratory failure, such as promoting centralization in high-volume hospitals, developing inter-hospital consultation systems, and implementing quality improvement programs in low-volume hospitals, according to the local healthcare infrastructure [28].

We observed the opposite effect on unfavorable outcomes and mortality in ICU admissions. One possible explanation is that patients treated in high-acuity settings may have a higher chance of survival, which unfortunately results in a higher risk of survival to discharge with new comorbidities [29]. Another explanation is that the severity of respiratory failure may not have been fully controlled for in the analysis, with ICU admissions potentially acting as a proxy for disease severity.

The present study has several limitations. First, the inclusion criterion for the use of ventilatory support may have selected a slightly different cohort from those identified by other criteria, such as specific respiratory status or oxygenation indices. The advent of high-flow nasal cannula therapy and its effect on the threshold for initiating respiratory support may have influenced our findings, although sensitivity analyses focusing on patients requiring invasive ventilation have yielded similar results. Second, our outcome measurement may have differed slightly from those using other scales, such as the Pediatric Overall Performance Category and Functional Status Scale [3, 5, 8]. In addition, owing to the nature of the Diagnosis Procedure Combination database, some components of unfavorable outcomes may not have been fully captured. However, we believe that our outcome measurement, based on the respiratory, feeding, neurological, and renal functions, reflected a significantly reduced quality of life, and the use of composite unfavorable outcomes was the best available approach in the study dataset. Third, as our dataset did not include information on indications for ventilatory support, we cannot exclude the possibility that older children requiring ventilatory support due to exacerbation of underlying neurological conditions triggered by acute respiratory diseases were more prone to unfavorable outcomes. However, a sensitivity analysis that excluded children with neurological/neuromuscular conditions also identified older age as a risk factor for unfavorable outcomes. Lastly, the lack of detailed data on physiological parameters, ventilatory support, laboratory results, and radiological findings, along with other potential confounders, such as socioeconomic status and analgesic/sedative use, limits the depth of our analysis [24]. Future research should address these issues.

## Conclusion

This study revealed that approximately 10% of children with acute respiratory failure experienced unfavorable outcomes, highlighting a considerable public health concern in the pediatric population. This concern has been exacerbated by the high incidence of respiratory failure in children. Our analysis identified early predictors of unfavorable outcomes, including older age, underlying conditions, pneumonia diagnosis, and low hospital volume. These findings warrant future efforts to improve acute care services for children at increased risk of unfavorable outcomes due to acute respiratory failure. The identified early predictors could aid in risk stratification and enhance the provision of acute care services to vulnerable pediatric patients.

## Supplementary Information

Supplementary Figure 1. Sensitivity analyses of patient distribution and outcomes by age in mechanically ventilated patients (n=20,365). N^a^ indicates neonates of ≤28 days old. This analysis included children who required invasive ventilation within the first three days of hospitalization. Unfavorable outcomes included in-hospital death, discharge with new comorbidities—specifically, tracheostomy, home ventilation or oxygen therapy, tube feeding at discharge or the day before, gastrostomy during hospitalization, worsened neurological status at discharge, and renal failure (TIF 420 KB)

Supplementary file2 (docx 33 KB)

## Data Availability

The datasets analyzed during the current study are not publicly available because of the study protocol but are available from the corresponding author upon reasonable request.

## Abbreviations

PICU: Pediatric intensive care unit
ICD-10: International classification of diseases 10th revision
COVID-19: Coronavirus disease. 2019
ICU: Intensive care unit
