# Supplementary figures and images for: Early predictors of unfavorable outcomes in pediatric acute respiratory failure
Source: J Intensive Care. 2024 Dec 2;12:50. doi: 10.1186/s40560-024-00763-x (PMC11610168; doi:10.1186/s40560-024-00763-x)

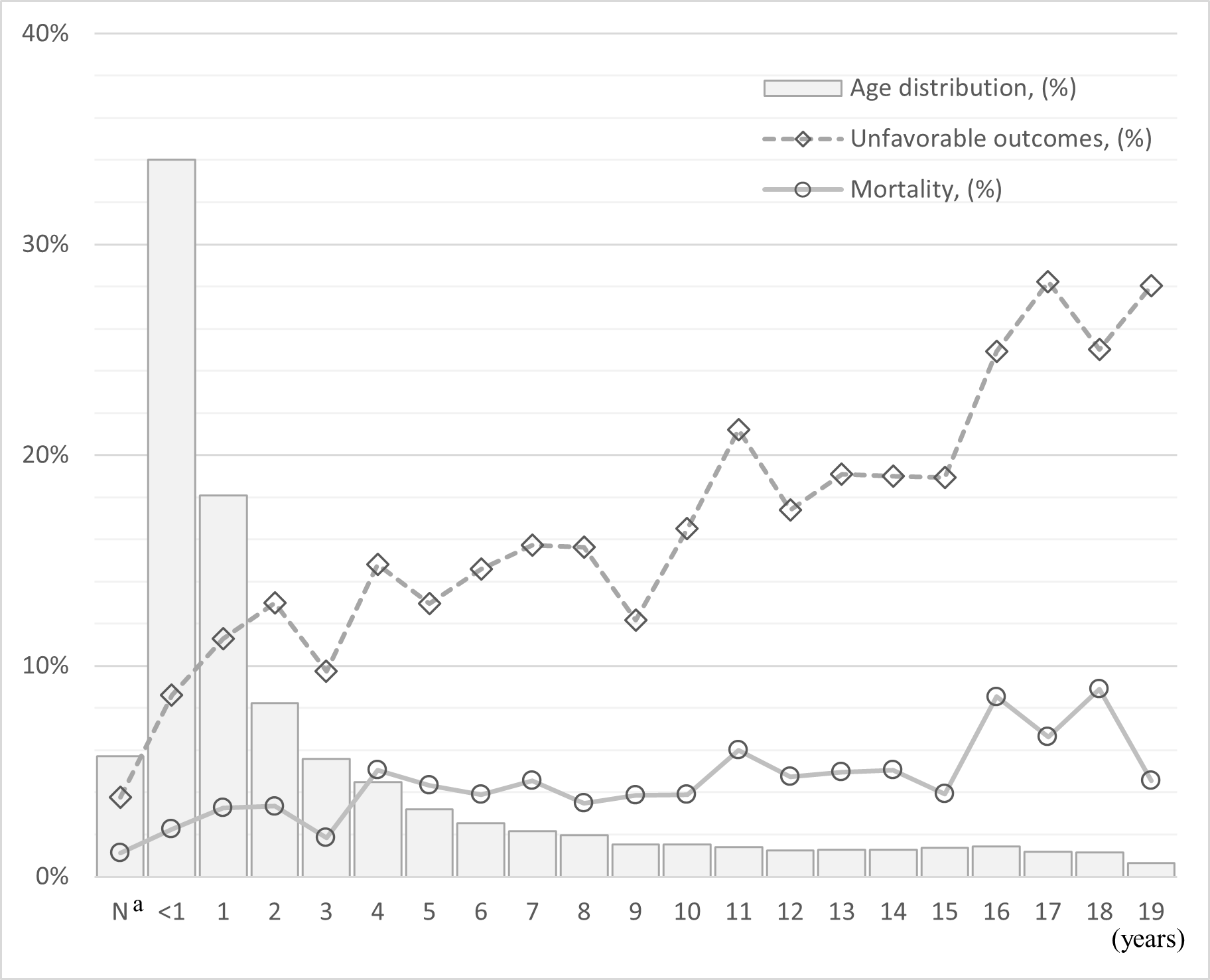

Supplement: Supplementary file 1 — Supplementary Figure 1. Sensitivity analyses of patient distribution and outcomes by age in mechanically ventilated patients (n=20,365). Na indicates neonates of ≤28 days old. This analysis included children who required invasive ventilation within the first three days of hospitalization. Unfavorable outcomes included in-hospital death, discharge with new comorbidities—specifically, tracheostomy, home ventilation or oxygen therapy, tube feeding at discharge or the day before, gastrostomy during hospitalization, worsened neurological status at discharge, and renal failure (TIF 420 KB) [file 40560_2024_763_MOESM1_ESM.tif]
