# Supplementary material for: Early predictors of unfavorable outcomes in pediatric acute respiratory failure
Source: J Intensive Care. 2024 Dec 2;12:50. doi: 10.1186/s40560-024-00763-x (PMC11610168; doi:10.1186/s40560-024-00763-x)
Supplement: Supplementary file 2 — Supplementary file2 (docx 33 KB) [file 40560_2024_763_MOESM2_ESM.docx]

Supplementary Table 1. Variance inflation factors in the primary regression models

| Variables | | VIF for unfavorable outcomes | VIF for death |
| --- | --- | --- | --- |
| Age | |  |  |
|  | <1 year | reference | |
|  | 1–5 years | 1.39 | 1.39 |
|  | 6–19 years | 1.45 | 1.45 |
| Underlying conditions | | 1.26 | 1.26 |
| Diagnostic category | |  |  |
|  | Bronchiolitis | reference | |
|  | Pneumonia | 1.65 | 1.65 |
|  | Asthma | 1.76 | 1.76 |
|  | Others | 1.09 | 1.09 |
| Pathogen | |  |  |
|  | Viral | reference | |
|  | Bacterial | 1.35 | 1.35 |
|  | Aspiration | 1.05 | 1.05 |
|  | COVID-19 | 1.01 | 1.01 |
|  | No detection | 1.78 | 1.78 |
| Organ dysfunction on admission | |  |  |
|  | Impaired consciousness |  |  |
|  | No-mild | reference | |
|  | Moderate | 1.05 | 1.05 |
|  | Severe | 1.16 | 1.16 |
|  | Acute liver failure | 1.04 | 1.04 |
|  | Thrombocytopenia/coagulopathy | 1.04 | 1.04 |
| Therapies within 3 days of admission | |  |  |
|  | Invasive ventilation | 1.22 | 1.22 |
|  | Renal replacement therapy | 1.13 | 1.13 |
|  | ECMO | 1.11 | 1.11 |
|  | Surgery | 1.01 | 1.01 |
|  | Cardiopulmonary resuscitation | 1.17 | 1.17 |
|  | Vasoactive drug | 1.30 | 1.30 |
|  | Corticosteroid | 1.21 | 1.21 |
| Transported from other hospitals | | 1.10 | 1.10 |
| Admitted to accredited ICUs | | 1.25 | 1.25 |
| Hospital volume | |  |  |
|  | 1–58 | 1.40 | 1.40 |
|  | 59–144 | 1.40 | 1.40 |
|  | 145–752 | reference | |

VIF, Variance inflation factor; COVID-19, coronavirus disease. 2019; ECMO, extracorporeal membrane oxygenation; ICU, intensive care unit.

Unfavorable outcomes included in-hospital death, discharge with new comorbidities—specifically, tracheostomy, home ventilation or oxygen therapy, tube feeding at discharge or the day before, gastrostomy during hospitalization, worsened neurological status at discharge, and renal failure.

Supplementary Table 2. Sensitivity analyses of early predictors in mechanically ventilated patients (n=20,365)

| Variables | | Unfavorable outcomes | | |  | In-hospital death | | |
| --- | --- | --- | --- | --- | --- | --- | --- | --- |
|  |  | Odds ratio (95% CI) | | *p* |  | Odds ratio (95% CI) | | *p* |
| Age | |  |  |  |  |  |  |  |
|  | <1 year | reference | |  |  | reference | |  |
|  | 1–5 years | 1.28 | (1.07–1.52) | <0.01 |  | 1.37 | (1.03–1.81) | 0.03 |
|  | 6–19 years | 1.48 | (1.25–1.74) | <0.01 |  | 1.16 | (0.85–1.58) | 0.34 |
| Underlying conditions | | 2.66 | (2.38–2.97) | <0.01 |  | 1.85 | (1.47–2.31) | <0.01 |
| Diagnostic category | |  |  |  |  |  |  |  |
|  | Bronchiolitis | reference | |  |  | reference | |  |
|  | Pneumonia | 2.09 | (1.80–2.42) | <0.01 |  | 2.34 | (1.65–3.30) | <0.01 |
|  | Asthma | 0.94 | (0.70–1.26) | 0.68 |  | 0.93 | (0.52–1.67) | 0.81 |
|  | Others | 0.76 | (0.48–1.21) | 0.25 |  | 0.64 | (0.23–1.77) | 0.39 |
| Pathogen | |  |  |  |  |  |  |  |
|  | Viral | reference | |  |  | reference | |  |
|  | Bacterial | 1.05 | (0.85–1.30) | 0.65 |  | 0.99 | (0.69–1.43) | 0.97 |
|  | Aspiration | 0.86 | (0.56–1.33) | 0.51 |  | 0.75 | (0.30–1.86) | 0.53 |
|  | COVID-19 | 0.90 | (0.31–2.64) | 0.85 |  | 1.58 | (0.28–8.94) | 0.60 |
|  | No detection | 1.60 | (1.38–1.86) | <0.01 |  | 1.63 | (1.22–2.19) | <0.01 |
| Organ dysfunction on admission | |  |  |  |  |  |  |  |
|  | Impaired consciousness |  |  |  |  |  |  |  |
|  | No-mild | reference | |  |  | reference | |  |
|  | Moderate | 1.12 | (0.91–1.40) | 0.29 |  | 1.94 | (1.29–2.91) | <0.01 |
|  | Severe | 1.00 | (0.87–1.16) | 0.96 |  | 2.33 | (1.81–3.02) | <0.01 |
|  | Acute liver failure | 2.80 | (0.59–13.37) | 0.20 |  | 1.42 | (0.32–6.39) | 0.65 |
|  | Thrombocytopenia/coagulopathy | 2.00 | (1.41–2.85) | <0.01 |  | 2.43 | (1.45–4.10) | <0.01 |
| Therapies within 3 days of admission | |  |  |  |  |  |  |  |
|  | Invasive ventilation |  | － |  |  |  | － |  |
|  | Renal replacement therapy | 2.21 | (1.29–3.79) | <0.01 |  | 3.45 | (1.72–6.89) | <0.01 |
|  | ECMO | 1.07 | (0.56–2.04) | 0.85 |  | 0.61 | (0.26–1.44) | 0.26 |
|  | Surgery | 3.48 | (2.08–5.82) | <0.01 |  | 0.83 | (0.23–2.99) | 0.78 |
|  | Cardiopulmonary resuscitation | 12.74 | (9.57–16.95) | <0.01 |  | 22.69 | (16.67–30.90) | <0.01 |
|  | Vasoactive drug | 2.60 | (2.21–3.06) | <0.01 |  | 9.51 | (7.35–12.30) | <0.01 |
|  | Corticosteroid | 1.02 | (0.91–1.15) | 0.75 |  | 0.85 | (0.67–1.08) | 0.18 |
| Transported from other hospitals | | 1.14 | (0.99–1.31) | 0.07 |  | 1.02 | (0.79–1.31) | 0.89 |
| Admitted to accredited ICUs | | 1.59 | (1.39–1.82) | <0.01 |  | 0.60 | (0.46–0.78) | <0.01 |
| Hospital volume | |  |  |  |  |  |  |  |
|  | 1–58 | 1.46 | (1.14–1.86) | <0.01 |  | 2.68 | (2.01–3.57) | <0.01 |
|  | 59–144 | 1.23 | (0.95–1.58) | 0.11 |  | 1.96 | (1.45–2.65) | <0.01 |
|  | 145–752 | reference | |  |  | reference | |  |

CI, confidence interval; COVID-19, coronavirus disease. 2019; ECMO, extracorporeal membrane oxygenation; ICU, intensive care unit.

Unfavorable outcomes included in-hospital death, discharge with new comorbidities—specifically, tracheostomy, home ventilation or oxygen therapy, tube feeding at discharge or the day before, gastrostomy during hospitalization, worsened neurological status at discharge, and renal failure.

In this multivariable analysis, 20,365 children who required invasive ventilation within the first three days of hospitalization were included.

Supplementary Table 3. Sensitivity analyses of early predictors after excluding patients with bronchiolitis (n= 20,856)

| Variables | | Unfavorable outcomes | | |  | In-hospital death | | |
| --- | --- | --- | --- | --- | --- | --- | --- | --- |
|  |  | Odds ratio (95% CI) | | *p* |  | Odds ratio (95% CI) | | *p* |
| Age | |  |  |  |  |  |  |  |
|  | <1 year | reference | |  |  | reference | |  |
|  | 1–5 years | 1.28 | (1.10–1.48) | <0.01 |  | 1.29 | (0.99–1.69) | 0.06 |
|  | 6–19 years | 1.40 | (1.21–1.63) | <0.01 |  | 1.09 | (0.81–1.48) | 0.57 |
| Underlying conditions | | 2.61 | (2.34–2.92) | <0.01 |  | 1.93 | (1.55–2.40) | <0.01 |
| Diagnostic category | |  |  |  |  |  |  |  |
|  | Bronchiolitis | － | |  |  | － | |  |
|  | Pneumonia | 2.17 | (1.74–2.69) | <0.01 |  | 2.47 | (1.58–3.88) | <0.01 |
|  | Asthma | reference | |  |  | reference | |  |
|  | Others | 0.75 | (0.46–1.22) | 0.25 |  | 0.68 | (0.24–1.89) | 0.46 |
| Pathogen | |  |  |  |  |  |  |  |
|  | Viral | reference | |  |  | reference | |  |
|  | Bacterial | 1.18 | (0.97–1.43) | 0.09 |  | 1.10 | (0.77–1.58) | 0.60 |
|  | Aspiration | 0.79 | (0.53–1.18) | 0.24 |  | 0.63 | (0.23–1.74) | 0.37 |
|  | COVID-19 | 1.07 | (0.42–2.73) | 0.89 |  | 1.62 | (0.31–8.35) | 0.57 |
|  | No detection | 1.75 | (1.51–2.03) | <0.01 |  | 1.67 | (1.24–2.25) | <0.01 |
| Organ dysfunction on admission | |  |  |  |  |  |  |  |
|  | Impaired consciousness |  |  |  |  |  |  |  |
|  | No-mild | reference | |  |  | reference | |  |
|  | Moderate | 1.20 | (0.98–1.47) | 0.08 |  | 2.13 | (1.44–3.14) | <0.01 |
|  | Severe | 1.05 | (0.91–1.21) | 0.51 |  | 2.43 | (1.88–3.14) | <0.01 |
|  | Acute liver failure | 2.49 | (0.49–12.62) | 0.27 |  | 1.71 | (0.36–8.16) | 0.50 |
|  | Thrombocytopenia/coagulopathy | 1.93 | (1.37–2.71) | <0.01 |  | 2.32 | (1.36–3.97) | <0.01 |
| Therapies within 3 days of admission | |  |  |  |  |  |  |  |
|  | Invasive ventilation | 1.48 | (1.24–1.76) | <0.00 |  | 3.20 | (1.77–5.80) | <0.00 |
|  | Renal replacement therapy | 1.96 | (1.13–3.41) | 0.02 |  | 2.94 | (1.47–5.86) | <0.01 |
|  | ECMO | 1.25 | (0.68–2.30) | 0.47 |  | 0.69 | (0.31–1.55) | 0.37 |
|  | Surgery | 4.01 | (2.37–6.80) | <0.01 |  | 0.84 | (0.24–2.93) | 0.78 |
|  | Cardiopulmonary resuscitation | 12.48 | (9.31–16.72) | <0.01 |  | 20.21 | (14.84–27.52) | <0.01 |
|  | Vasoactive drug | 2.55 | (2.17–3.01) | <0.01 |  | 8.91 | (6.92–11.49) | <0.01 |
|  | Corticosteroid | 0.96 | (0.86–1.08) | 0.51 |  | 0.80 | (0.63–1.03) | 0.09 |
| Transported from other hospitals | | 1.17 | (1.02–1.34) | 0.03 |  | 1.04 | (0.81–1.35) | 0.75 |
| Admitted to accredited ICUs | | 1.54 | (1.35–1.75) | <0.01 |  | 0.62 | (0.48–0.82) | <0.01 |
| Hospital volume | |  |  |  |  |  |  |  |
|  | 1–58 | 1.46 | (1.13–1.88) | <0.01 |  | 2.42 | (1.80–3.25) | <0.01 |
|  | 59–144 | 1.21 | (0.93–1.57) | 0.15 |  | 1.76 | (1.29–2.42) | <0.01 |
|  | 145–752 | reference | |  |  | reference | |  |

CI, confidence interval; COVID-19, coronavirus disease. 2019; ECMO, extracorporeal membrane oxygenation; ICU, intensive care unit.

Unfavorable outcomes included in-hospital death, discharge with new comorbidities—specifically, tracheostomy, home ventilation or oxygen therapy, tube feeding at discharge or the day before, gastrostomy during hospitalization, worsened neurological status at discharge, and renal failure.

Supplementary Table 4. Sensitivity analyses of early predictors after excluding patients with underlying neurological/neuromuscular conditions (n= 25,436)

| Variables | | Unfavorable outcomes | | |  | In-hospital death | | |
| --- | --- | --- | --- | --- | --- | --- | --- | --- |
|  |  | Odds ratio (95% CI) | | *p* |  | Odds ratio (95% CI) | | *p* |
| Age | |  |  |  |  |  |  |  |
|  | <1 year | reference | |  |  | reference | |  |
|  | 1–5 years | 1.52 | (1.27–1.82) | <0.01 |  | 1.32 | (1.00–1.74) | 0.05 |
|  | 6–19 years | 1.75 | (1.44–2.12) | <0.01 |  | 1.38 | (0.98–1.94) | 0.06 |
| Underlying conditions | | 2.57 | (2.27–2.91) | <0.01 |  | 2.85 | (2.21–3.68) | <0.01 |
| Diagnostic category | |  |  |  |  |  |  |  |
|  | Bronchiolitis | reference | |  |  | reference | |  |
|  | Pneumonia | 1.86 | (1.59–2.18) | <0.01 |  | 2.36 | (1.59–3.49) | <0.01 |
|  | Asthma | 0.76 | (0.58–1.00) | 0.05 |  | 0.67 | (0.34–1.32) | 0.25 |
|  | Others | 0.60 | (0.36–1.02) | 0.06 |  | 0.58 | (0.17–1.94) | 0.38 |
| Pathogen | |  |  |  |  |  |  |  |
|  | Viral | reference | |  |  | reference | |  |
|  | Bacterial | 1.25 | (0.99–1.57) | 0.06 |  | 1.16 | (0.77–1.74) | 0.48 |
|  | Aspiration | 1.00 | (0.69–1.45) | 0.98 |  | 0.80 | (0.30–2.15) | 0.65 |
|  | COVID-19 | 0.79 | (0.28–2.17) | 0.64 |  | 1.80 | (0.29–11.03) | 0.53 |
|  | No detection | 1.88 | (1.59–2.23) | <0.01 |  | 1.87 | (1.34–2.60) | <0.01 |
| Organ dysfunction on admission | |  |  |  |  |  |  |  |
|  | Impaired consciousness |  |  |  |  |  |  |  |
|  | No-mild | reference | |  |  | reference | |  |
|  | Moderate | 1.30 | (1.04–1.63) | 0.02 |  | 2.38 | (1.51–3.74) | <0.01 |
|  | Severe | 0.99 | (0.83–1.19) | 0.95 |  | 2.45 | (1.80–3.36) | <0.01 |
|  | Acute liver failure | 2.28 | (0.24–21.41) | 0.47 |  | 0.88 | (0.12–6.75) | 0.90 |
|  | Thrombocytopenia/coagulopathy | 1.77 | (1.17–2.67) | <0.01 |  | 3.21 | (1.75–5.87) | <0.01 |
| Therapies within 3 days of admission | |  |  |  |  |  |  |  |
|  | Invasive ventilation | 1.55 | (1.29–1.88) | <0.00 |  | 4.00 | (2.14–7.49) | <0.00 |
|  | Renal replacement therapy | 1.83 | (0.98–3.40) | 0.06 |  | 3.73 | (1.85–7.54) | <0.01 |
|  | ECMO | 1.27 | (0.63–2.57) | 0.50 |  | 0.44 | (0.17–1.16) | 0.10 |
|  | Surgery | 2.61 | (1.42–4.82) | <0.01 |  | 0.85 | (0.22–3.29) | 0.81 |
|  | Cardiopulmonary resuscitation | 14.04 | (10.31–19.12) | <0.01 |  | 35.07 | (25.03–49.14) | <0.01 |
|  | Vasoactive drug | 2.81 | (2.38–3.32) | <0.01 |  | 8.19 | (6.09–11.02) | <0.01 |
|  | Corticosteroid | 0.97 | (0.85–1.10) | 0.64 |  | 0.81 | (0.62–1.06) | 0.12 |
| Transported from other hospitals | | 1.12 | (0.95–1.33) | 0.19 |  | 1.01 | (0.75–1.35) | 0.95 |
| Admitted to accredited ICUs | | 1.39 | (1.20–1.61) | <0.01 |  | 0.59 | (0.43–0.80) | <0.01 |
| Hospital volume | |  |  |  |  |  |  |  |
|  | 1–58 | 1.44 | (1.07–1.93) | 0.02 |  | 2.79 | (1.95–4.01) | <0.01 |
|  | 59–144 | 1.19 | (0.88–1.61) | 0.25 |  | 2.01 | (1.37–2.96) | <0.01 |
|  | 145–752 | reference | |  |  | reference | |  |

CI, confidence interval; COVID-19, coronavirus disease. 2019; ECMO, extracorporeal membrane oxygenation; ICU, intensive care unit.

Unfavorable outcomes included in-hospital death, discharge with new comorbidities—specifically, tracheostomy, home ventilation or oxygen therapy, tube feeding at discharge or the day before, gastrostomy during hospitalization, worsened neurological status at discharge, and renal failure.

In this multivariable analysis, 3,926 children with underlying neuromuscular conditions were excluded.
